# Unilateral ‘cheetah-like’ endotheliopathy: A rare endothelial phenotype regressing after phacoemulsification

**DOI:** 10.1016/j.ajoc.2026.102666

**Published:** 2026-09-23

**Authors:** Sylvain Poinard, Oliver Dorado-Cortez, Zhiguo He, Fabien Forest, Daria-Andrada Onitiu, Philippe Gain, Gilles Thuret

**Affiliations:** a Ophthalmology Department, University Hospital, Saint-Etienne, France; b Laboratory Biology, Engineering and Imaging for Ophthalmology, BiiO, Faculty of Medicine, Health Innovation Campus, University Jean Monnet, Saint-Etienne, France; c Pathology Department, University Hospital, Saint-Etienne, France; d Genetic Department, University Hospital, Saint-Etienne, France

## Abstract

**Purpose:**

To report an exceptional case of unilateral endothelial corneal disease with unique specular microscopy findings and a regressive course following cataract surgery.

**Methods:**

We present a 60-year-old patient with a unilateral corneal anomaly discovered during a routine slit-lamp examination. Specular microscopy, retro-illumination (innovative prototype) were used for imaging. Endothelial cell density (ECD) was assessed, and genetic testing for TCF4 gene mutations was performed. During the follow-up, the patient underwent phacoemulsification for cataract surgery.

**Results:**

Retroillumination images showed numerous vesicular-like lesions in the center of the cornea that were not confluent. Specular microscopy revealed a cheetah-like pattern of dark spots centered in endothelial cells with normal ECD. Post-surgery, most vesicular-like lesions disappeared, replaced by regular pigment deposits, which diminished over time. ECD decreased by 49% one year post-surgery, but corneal transparency and normal thickness were maintained. Genetic testing for TCF4 triplet repeat expansions was negative.

**Conclusion:**

These findings reveal a previously uncharacterized endothelial anomaly, representing a diagnostic challenge given its strictly unilateral presentation and sudden resolution after phacoemulsification. The disappearance of vesicular-like lesions and subsequent pigment deposition suggest a possible relationship with membrane structures, such as macrovesicles or primary cilia. The findings highlight the value of retro-illumination imaging, which we have recently reintroduced using an innovative prototype. This technique proved crucial in detecting subtle and widespread endothelial abnormalities that were otherwise difficult to observe with a slit lamp, offering a valuable diagnostic tool for diagnosing and monitoring endothelial disorders.

## 1 Introduction

The corneal endothelium is a monolayer of flat, contiguous endothelial cells lining the posterior surface of the cornea. It plays an essential role in maintaining transparency, thanks to its barrier and pumping properties. It can be affected by a large variety of pathological processes, acquired or congenital, uni or bilateral, with or without a genetic component.

We report a case of exceptional unilateral endothelial disease with a particular appearance under specular microscopy and a curious regressive course after cataract surgery. After reviewing the literature, we will discuss the pathophysiological hypotheses.

## 2 Case report

We present the case of a 60-year-old patient whose corneal anomaly was discovered fortuitously during a routine visit. It concerned the left eye with mild amblyopia due to congenital astigmatism, with a baseline best-corrected visual acuity (BCVA) of 20/40. During the follow-up, this anomaly appeared to be strictly unilateral, non-progressive and involved the endothelial layer only.

The cornea was transparent under slit lamp and retroillumination (prototype of a far-red, high-resolution, glare-free retroilluminator^1^) showed innumerable vesicular-like lesions evenly distributed in the central 6 mm. Non-contact specular microscopy was unusual: The endothelial cell density (ECD) was normal (2778 cells/mm^2^) with a black spot in front of almost every cell given a cheetah-like pattern (Fig. 1). OCT thickness mapping and examination of the other eye were normal. A search for expansion of CTG triplet repeats in the TCF4 gene (present in 71% of patients in our team's series^2^) was performed on peripheral blood using TP and STR PCR according to a conventional technique.^3^

**Fig. 1:**
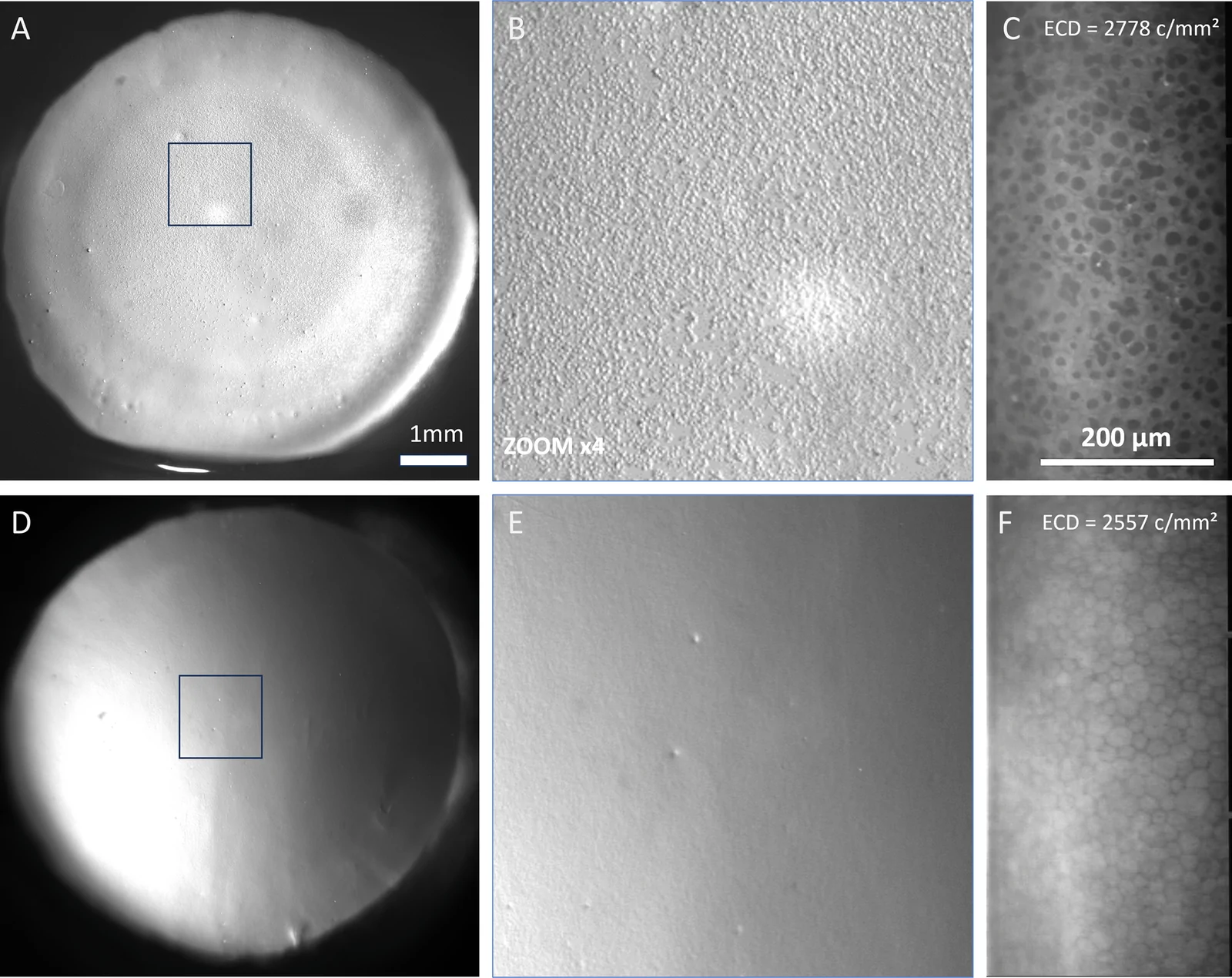
Retro-illumination (home-made prototype) of the diseased left cornea. The concentric rings were caused by a dense nuclear cataract. The zoom showed the thousands of regular vesicular-like lesions (B). Non-contact specular microscopy of this pathological cornea showing round dark spots (Cheetah-like) mostly isolated and centered in each endothelial cell and rarely coalescent (C). For comparison, Fig. 1D and E and F show the same exams for the fellow eye.

Four years after the discovery of the anomaly, the endothelial lesions remained stable, but the patient reported a subjective visual degradation accompanied by disabling photophobia, secondary to the progression of a dense corticonuclear cataract. At this point, the BCVA had dropped to 20/100. Despite the underlying amblyopia, the patient underwent an uneventful phacoemulsification and a 3-piece intraocular lens (IOL) was implanted in the capsular bag. The total surgical time was 13 minutes, with a total ultrasound time of 1 minute and 29 seconds and a cumulative dissipated energy (CDE) of 17.86 (Constellation Vision System, Alcon, Geneva, Switzerland). At the end of the surgery, almost all the vesicular-like lesions had disappeared. The cornea was oedematous on day 1 but regained its transparency within 7 days, and the best-corrected visual acuity returned to 20/40.

The lesions in retroillumination and specular microscopy did not reappear, but were replaced by pigment deposits, which were also very regular and diminished over time (Fig. 2). After 1 year, the ECD was 1400 cells/mm^2^ (-49%), the cornea transparent and of normal thickness. The patient did not exhibit any expansion of triplet repeats in the TCF4 gene. (Fig. 3).

**Fig. 2:**
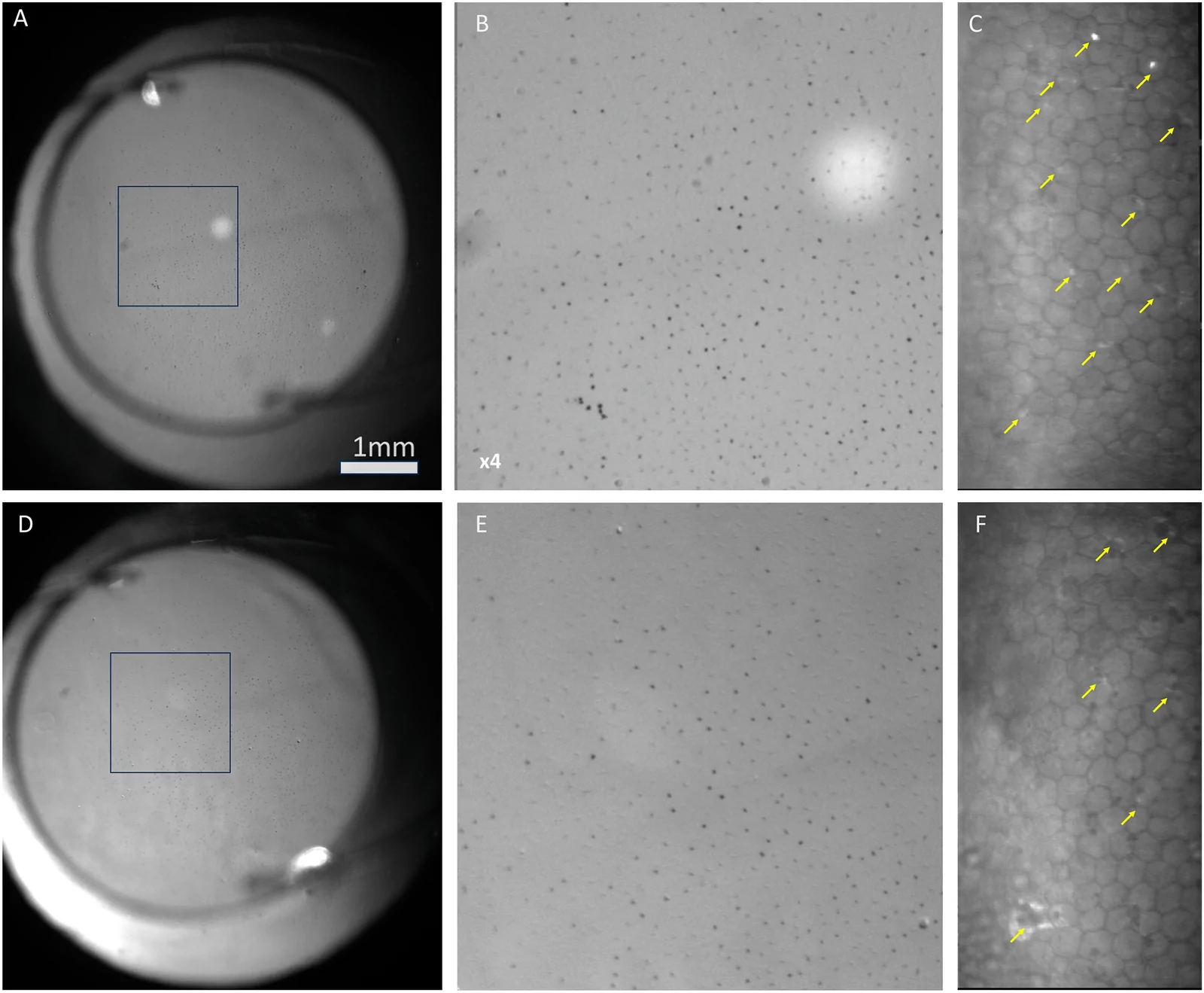
Retro-illuminated images (A, B) of the same affected cornea 4 weeks after phacoemulsification: disappearance of the initial vesicular-like lesions, replaced by pigments. With specular microscopy (C) the pigments were hardly visible as hyper-reflective white spot (yellow arrow). Some cells showed black micro-spots that appeared to be residues from the initial drops. Endothelial cell density: 1454 cells/mm^2^. A year later the pigments seem less visible (D, E, F). (For interpretation of the references to colour in this figure legend, the reader is referred to the Web version of this article.)

**Fig. 3:**
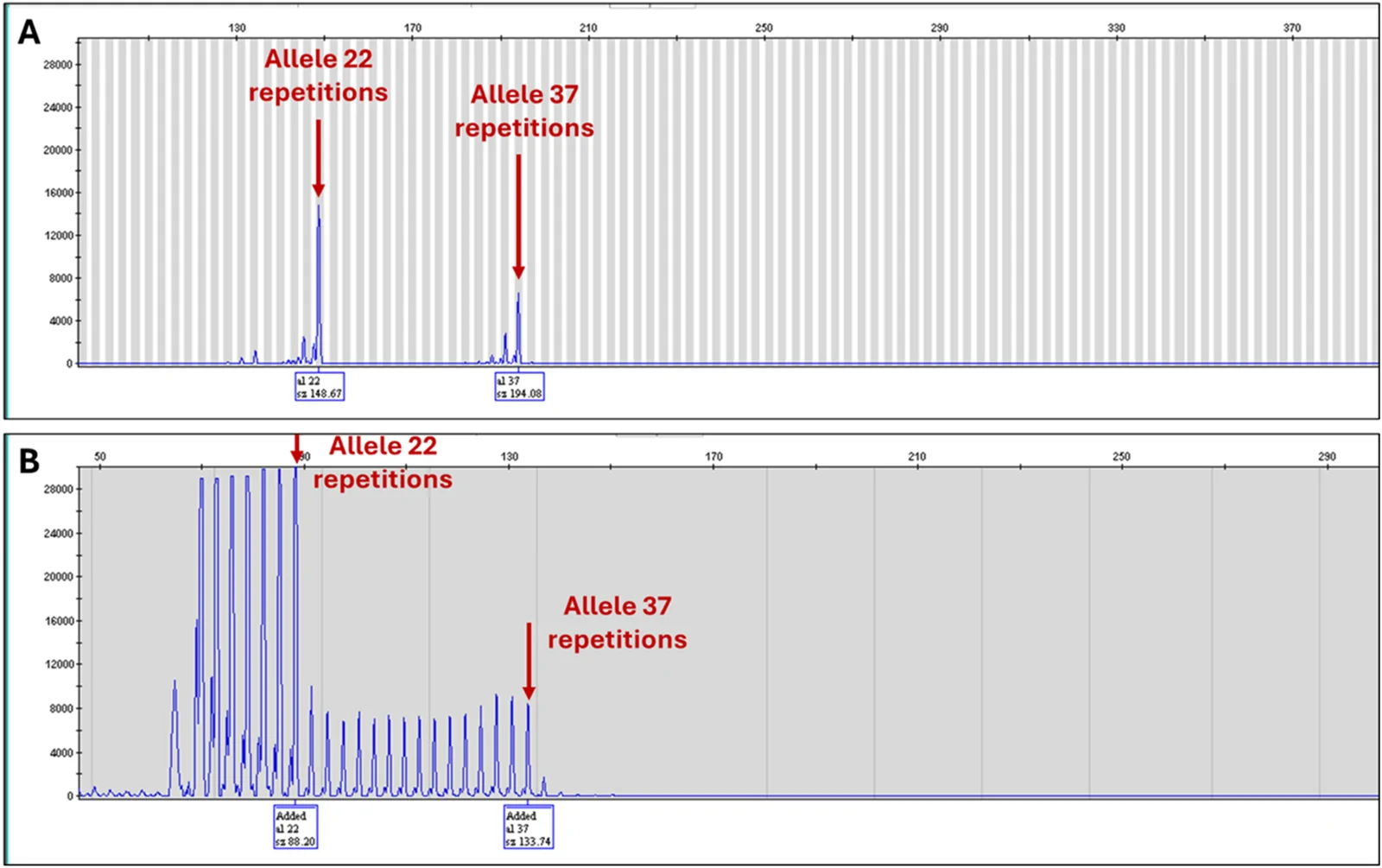
Triplet repeat in the TCF4 gene investigated by STR-PCR (A) and TP-PCR (B).

## 3 Discussions

We found only one similar case in the literature, published by Smedowski et al., describing a 36-year-old patient with almost identical *in vivo* confocal microscopy images of vesicle-like endothelial lesions with a regressive course and followed by an endothelial cell loss.^4^ Whether these two clinical presentations share an identical molecular etiology remains an open question. Indeed, their case was bilateral and asymmetric, occurring in a patient who had not undergone surgery and who exhibited a spontaneous regressive course. Notably, specular microscopy was not performed in their report, and cellular characterization relied exclusively on confocal microscopy. Due to this bilateral presentation and the absence of any other identifiable cause, the authors hypothesized an unknown form of corneal dystrophy.

The term “corneal dystrophy” is reserved for a heterogeneous group of corneal conditions that generally have the following characteristics: hereditary, non-inflammatory, typically bilateral, slowly progressive that do not have systemic associations.^5^ Although the appearance of the lesions under retroillumination closely resembles guttae in Fuchs' endothelial dystrophy, the particular appearance under specular microscopy, the unilateral nature and the regression of the lesions after surgery led us to be cautious about using the term ‘guttae’ given the uncertainty of their nature, and we preferred the term ‘vesicular-like lesions’. Similarly, it seemed inappropriate to us to use the term “dystrophy” to characterize this condition. The triplet repeat in the TCF4 gene (involved in 70% of FECD in Caucasians and probably in other corneal disorders) was also investigated in this patient and found to be negative.

Posterior polymorphous corneal dystrophy (PPMD) was also considered as a differential diagnosis. However, several clinical and morphological features rule it out. Morphologically, the vesicular or band-like lesions of PPMD are macroscopic structural alterations that span across multiple cell borders, disrupting the regular endothelial mosaic, and are characterized on specular microscopy by an abnormal, epithelial-like cell population. In contrast, our patient exhibited an intact endothelial mosaic where each individual cell contained a single, centered, vesicle-like structure. Furthermore, PPMD is a bilateral genetic condition, whereas extensive evaluation of the patient's fellow eye using both high-magnification retroillumination and specular microscopy confirmed a completely normal endothelial monolayer. Finally, the permanent structural modifications of Descemet's membrane in PPMD are incompatible with the instantaneous intraoperative clearance observed here.

Among the causes of non-dystrophic endotheliopathy, the “ICE” (irido-corneal endothelial) syndromes are a group of unilateral disorders of the corneal endothelium. They are characterized by the presence of an abnormal population of endothelial cells that produce a basal neomembrane (neo-Descemet) whose retractile properties are responsible for progressive iris anomalies (corectopia) and increase of the intra ocular pressure due to trabecular meshwork obstruction. In our case, the iris was intact and intraocular pressure was normal. Although conventional specular microscopy did not reveal any abnormal overlap of cell populations suggestive of ICE syndrome, *in vivo* confocal microscopy (IVCM) would have allowed for a more precise cellular characterization, particularly for early or incomplete stages. We were unable to perform this examination, which constitutes a limitation of this study. However the appearance of the lesions on specular microscopy and their regression remain atypical compared to the clinical variants of this syndrome.^6^

Several arguments do not support a viral endotheliitis origin despite the unilateral nature: 1/No active inflammation was ever observed during any of the visits, with the lesions remaining stable for several years prior to surgery 2/The abnormalities disappeared at the end of the surgery when the viscoelastic was washed out, and not after the introduction of steroids in the postoperative period, which could have had an effect on a supposed endotheliitis.

Given the strictly unilateral nature of the disease and its stability over time, its association with congenital astigmatism, and the resulting amblyopia, a localized developmental or vascular anomalous event during anterior segment embryogenesis cannot be excluded. A congenital dysgenesis of the neural crest-derived endothelial cells might have predisposed this specific eye to later manifest this unique vesicular phenotype. The regular arrangement of dark spots on each cell and their disappearance on washing suggest that this is a membrane structure, possibly a macrovesicle or a structure related to the primary cilia.^7^ Their origin remains unclear, as does that of the pigments and their regression over time. Nevertheless, one of the explanations for these postoperative pigments could be the cataract surgery itself, which may have promoted the shedding and dispersing of iris pigment, especially with the use of a 3-piece intraocular lens. The instantaneous clearance of these vesicular-like lesions during automated irrigation/aspiration of the viscoelastic device provides a key pathophysiological insight. Unlike true corneal guttae observed in Fuchs dystrophy—which represent permanent, irreversible extracellular collagenous deposits secreted into Descemet's membrane—these structures exhibited mechanical or fluidic instability. Two hypotheses can explain this sudden intraoperative resolution. First, the lesions may represent fragile, fluid-filled intracellular macrovesicles or transient intercellular edema protruding into the anterior chamber, ruptured by the high-flow fluidics and mechanical shear stress of the irrigation/aspiration tip. Second, the cohesive nature of the viscoelastic may have exerted a mechanical or oncotic pressure that collapsed these delicate protrusions, which were then cleared during the final washout. This structural vulnerability supports our decision to avoid the term ‘guttae’ and points toward a reversible, non-dystrophic cellular event.

Although the literature presents variable results due to numerous risk factors (age, initial DCE, anterior chamber depth, cataract density, phacoemulsification technique), the cell loss observed after phacoemulsification of a dense cataract is generally estimated at around 10% at one year.^8^ In our case, there was significant endothelial loss despite the standard technique used. This suggests that the presence of vesicles and their destruction may have had an impact on the survival of corneal endothelial cells.

This clinical case illustrates the value of retro-illumination imaging, which we have recently brought back into use.^1^ We acknowledge that our custom-made, far-red device is currently a non-commercial prototype. While standard clinical slit-lamps equipped with high-resolution digital cameras and decoupled illumination can approximate this view, conventional setups typically require capturing multiple acquisition angles to image the entire cornea while successfully avoiding the slit-lamp light reflex. In contrast, our method allows for a complete, single-shot, and glare-free exploration of the central cornea, covering the entire diameter of the dilated pupil. It thus becomes possible to detect and monitor subtle, widespread abnormalities that are difficult to observe reproducibly with a standard slit lamp. This opens new perspectives for the early diagnosis and monitoring of various corneal pathologies, as we previously demonstrated in a study illustrating the peripheral involvement of Fuchs' dystrophy through the presence of corneal striae around 360°.^9^

## CRediT authorship contribution statement

**Sylvain Poinard:** Writing – review & editing, Writing – original draft, Investigation, Conceptualization. **Oliver Dorado-Cortez:** Writing – original draft, Investigation. **Zhiguo He:** Investigation. **Fabien Forest:** Writing – original draft, Investigation. **Daria-Andrada Onitiu:** Writing – original draft, Investigation. **Philippe Gain:** Project administration. **Gilles Thuret:** Writing – review & editing, Writing – original draft, Validation, Investigation, Conceptualization.

## Ethics declaration

Written informed consent to take part in the study and to publish the article has been obtained from all participants or their legal representatives. The privacy rights of participants have been observed.

This study was performed in compliance with relevant laws, regulatory frameworks and guidelines where the research took place. Ethics committee approval was not required under relevant laws and institutional guidelines. Ethics approval was not required by the local institutional review board for the publication of this case report. Written informed consent was obtained from the patient for the publication of this case report and any accompanying images.

This research follows the CARE guidelines and the CARE checklist.

## Patient consent

Written informed consent was obtained from the patient for publication of this case report and any accompanying images.

## Authorship

All authors attest that they meet the current ICMJE criteria for Authorship.

## Funding

No funding or grant support

## Declaration of competing interest

The authors declare that they have no known competing financial interests or personal relationships that could have appeared to influence the work reported in this paper.
